# A Teaching Case: Persistent COVID-19 Pneumonia Resembling Cryptogenic Organizing Pneumonia in a Patient With Remitted Lymphoma

**DOI:** 10.7759/cureus.48319

**Published:** 2023-11-05

**Authors:** Toyoshi Yanagihara, Masako Kadowaki, Junji Otsuka, Akiko Ishimatsu, Kazuhito Taguchi, Yuki Moriuchi, Hiroaki Ogata, Atushi Moriwaki, Makoto Yoshida

**Affiliations:** 1 Department of Respiratory Medicine, National Hospital Organization Fukuoka National Hospital, Fukuoka, JPN; 2 Department of Infectious Diseases, National Hospital Organization Fukuoka National Hospital, Fukuoka, JPN

**Keywords:** sars-cov-2, bronchoalveolar lavage fluid, follicular lymphoma, cryptogenic organizing pneumonia, covid-19

## Abstract

We report a case of a female patient in her 50s, previously diagnosed with follicular lymphoma (now in complete remission), who was admitted to our hospital due to antibiotic-resistant pneumonia lasting a month. The patient had contracted coronavirus disease 2019 (COVID-19) pneumonia a year earlier and exhibited persistent hypogammaglobulinemia. Chest CT scans revealed wondering ground-glass opacities and consolidations initially suggestive of cryptogenic organizing pneumonia (COP). Despite repeatedly negative nasopharyngeal SARS-CoV-2 tests, the virus was detected in the bronchoalveolar lavage fluid (BALF) using the BioFire FilmArray Respiratory Panel 2.1. She was subsequently diagnosed with COVID-19 pneumonia and responded well to treatment with remdesivir (RDV) and intravenous immunoglobulin. The SARS-CoV-2 variant in the BALF was suspected as the Omicron variant (XBB.1.16), prevalent in the area at the admission, indicating a re-infection rather than a recurrence. This case underscores the protracted nature of COVID-19 pneumonia in immunocompromised patients and the risks of false negatives in nasopharyngeal SARS-CoV-2 tests. Direct SARS-CoV-2 measurement from BALF can be crucial in such cases. A COP diagnosis based solely on imaging and administering corticosteroids without antiviral treatment might exacerbate the situation by reactivating SARS-CoV-2. Given the current pandemic, clinicians should be aware of the potential for persistent or recurrent COVID-19, particularly in immunocompromised patients.

## Introduction

Immunocompromised patients have a recognized increased risk of persistent or recurrent coronavirus disease 2019 (COVID-19) due to challenges in achieving adequate viral clearance, as evidenced by numerous studies [1-12]. Diagnostic and treatment difficulties emerge when these patients present with persistent lung infections that mimic the characteristics of organizing pneumonia on radiological imaging. Organizing pneumonia is an interstitial lung disease subset that manifests as an inflammatory and reparative process in the lung tissue [13]. This response stems from fibroblast proliferation in peripheral air spaces without undermining the lung's structural integrity. The idiopathic variant of this condition is termed cryptogenic organizing pneumonia (COP), while other forms arise from infections, drug reactions, and connective tissue diseases [13]. The diagnostic challenge deepens when nasopharyngeal coronavirus antigens or PCR tests turn negative, potentially prompting clinicians to initiate corticosteroid treatments for suspected COP. This approach could inadvertently exacerbate pneumonia by reactivating the virus in immunocompromised patients. We experienced a patient who manifested persistent COVID-19 pneumonia, initially resembling COP, with negative results of nasopharyngeal SARS-CoV-2 tests. This case highlights the details of managing immunocompromised patients with COVID-19 and accentuates the crucial nature of accurate diagnosis and careful treatment to avoid severe complications.

## Case presentation

**Figure 1:**
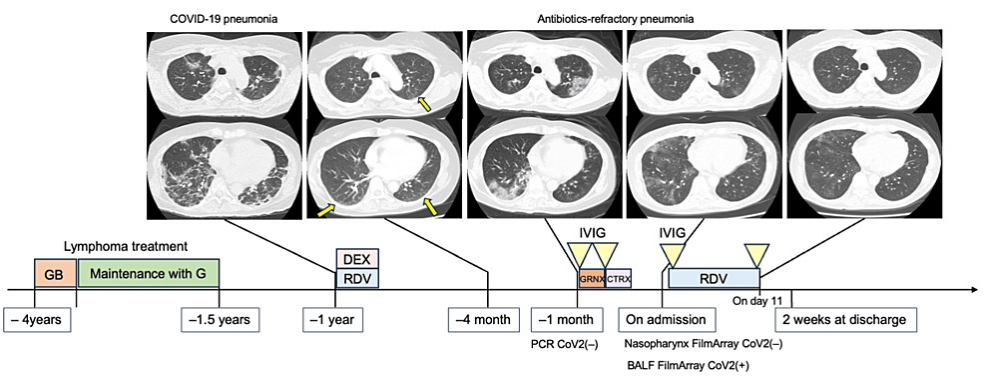
The clinical course of the patient. The patient was diagnosed with follicular lymphoma four years earlier. After four courses of GB therapy (obinutuzumab and bendamustine) with complete remission, she underwent a two-year maintenance regimen with obinutuzumab, the last dose given one and a half years earlier. One year earlier, she developed COVID-19 pneumonia. Follow-up CT scans four months earlier showed residual faint ground-glass opacities (arrows). One month earlier, she developed antibiotics-refractory, persistent pneumonia, resulting in her transfer to our institution. She was diagnosed with COVID-19 pneumonia based on the positive result for SARS-CoV-2 in the BALF by the FilmArray Respiratory Panel 2.1. She was treated with RDV and IVIG supplementation, resulting in her recovery. G, obinutuzumab; DEX, dexamethasone; GRNX, garenoxacin; CTRX, ceftriaxone; BALF, bronchoalveolar lavage fluid; RDV, remdesivir; IVIG, intravenous immunoglobulin

A female patient in her 50s, with a history of well-managed type 2 diabetes, was diagnosed with follicular lymphoma (Grade 2, clinical stage IV, FLIPI Int, FLIP2 High) four years earlier. After four courses of GB therapy (obinutuzumab and bendamustine) with complete remission, she underwent a two-year maintenance regimen with obinutuzumab, the last dose given one and a half years earlier (Figure 1). After that, she was monitored without active treatment. One year earlier, she presented with COVID-19 symptoms such as fatigue, diarrhea, and anorexia, which required hospitalization. Concurrently, she was diagnosed with COVID-19 pneumonia and recovered with remdesivir (RDV) (an initial dose of 200 mg followed by daily doses of 100 mg) and dexamethasone, leading to her discharge. Follow-up CT scans nine and four months earlier showed residual faint ground-glass opacities.

**Figure 2:**
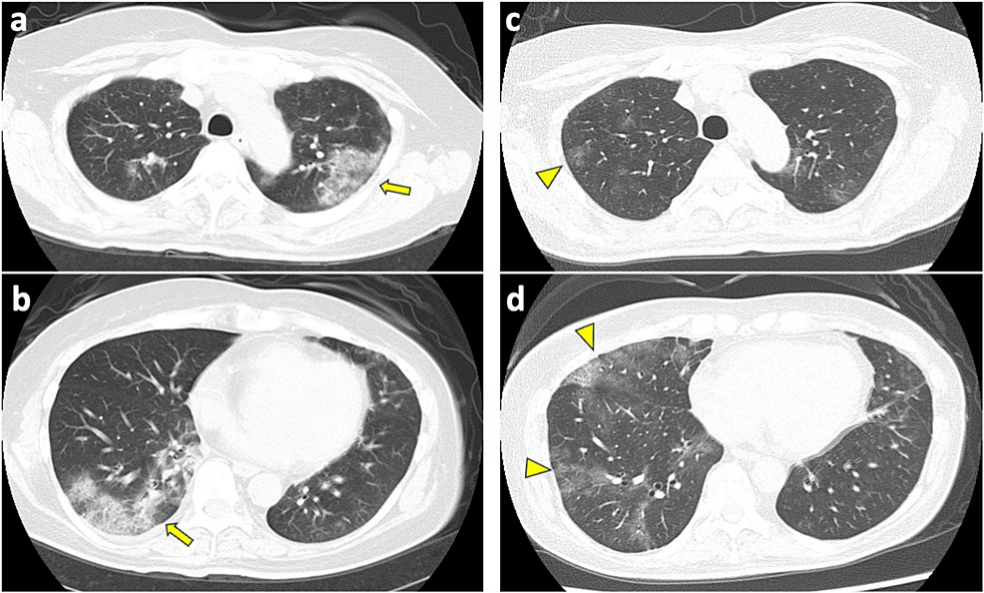
Chest CT images of the patient one month earlier and on admission. Chest CT images of the patient showing (a and b) patchy ground glass with consolidation opacities with reversed halo signs (arrows) in both lungs one month earlier and (c and d) wondering patchy ground glass with consolidation (arrowheads) on admission.

A month earlier, she presented with fever, general fatigue, and exertional dyspnea (mMRC Grade 3). Initial treatment with garenoxacin for presumed acute pneumonia at another facility failed to improve her condition, necessitating hospitalization. Administration of intravenous ceftriaxone did not alleviate her persistent symptoms, including fever, cough, and fatigue. Repeated CT scans showcasing persistent ground-glass opacities led to a COP suspicion, resulting in her transfer to our institution (Figure 2). Her regular medication included fluconazole and trimethoprim-sulfamethoxazole.

At admission on September 2023, vital signs and physical examination included a body temperature of 36.9°C (with routine loxoprofen administration), SpO_2_ of 98% on room air, respiratory rate of 24/min, pulse rate of 120/min, and blood pressure of 105/71 mmHg. Mild late inspiratory crackles were evident bilaterally on the back, but no cardiac murmurs, edema, or rashes were observed. Laboratory findings revealed WBC of 5,240/uL, Hb of 9.5g/dL, PLT of 332x10^3^/uL, CRP of 2.0 mg/dL, LDH of 255 U/L, and KL-6 of 747 U/mL (reference: <500 U/mL). Hypogammaglobulinemia was observed with a decrease of each component as follows: IgG at 535 mg/dL (reference: 861-1747), IgA at 48 mg/dL (reference: 93-393), and IgM at 18 mg/dL (reference: 50-269). Tests were negative for beta-D-glucan, cytomegalovirus antigenemia, T-SPOT.TB, anti-nuclear antibodies, myeloperoxidase (MPO) anti-neutrophil cytoplasmic antibodies (ANCA), proteinase 3 (PR3)-ANCA, and anti-aminoacyl-tRNA synthetase (ARS) antibodies. Nasopharyngeal SARS-CoV-2 antigen (HISCL™ SARS-CoV-2 antigen test) and PCR tests (Smart Gene® SARS-CoV-2 detection reagent), along with BioFire FilmArray Respiratory Panel 2.1, were all negative. CT scans displayed multiple patchy ground-glass opacities and consolidations in both lungs (Figure 2).

**Figure 3:**
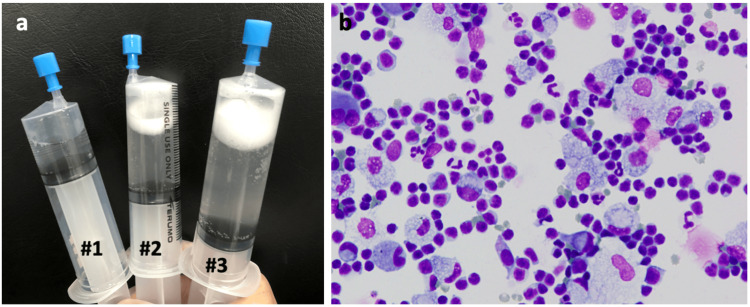
BAL fluid of the patient. (a) BAL from the right B4 retrieved 77/150 mL of slightly turbid white fluid. (b) May-Giemsa staining of BALF cells showing lymphocytosis (85% of lymphocytes). BAL, bronchoalveolar lavage

Given the antibiotic-refractory lung infiltrates and differential diagnoses of COP or persistent COVID-19 pneumonia, a bronchoscopy was performed on the second day of admission. Bronchoalveolar lavage (BAL) from the right B5 yielded 77/150 mL of slightly turbid white fluid (Figure 3a). Subsequent transbronchial lung biopsy (TBLB) was performed from the right B2b, B3a, and B8a. The BALF analysis revealed elevated cell count (6.1x10^5^/mL) and lymphocytosis with 85% lymphocytes, 14% macrophages, 0.5% neutrophils, and 0.3% eosinophils (Figure 3b).

Flow cytometric analysis of BALF showed a decrease in CD4/CD8 ratio of 0.11, with 72.8% HLA-DR+CD3+ within the CD3+ population and 0.7% CD25+CD4+ within the CD4+ population. The BALF showed no detectable pathogenic bacteria. Although the SARS-CoV-2 antigen test was negative, the FilmArray Respiratory Panel 2.1 identified SARS-CoV-2 in the BALF, further confirmed by the Smart Gene® PCR with a cycle threshold value of 39. The BALF sample underwent further analysis at a public health center using the QiaSEQ FX protocol (Qiagen, version 1.4) for sequencing, and the analysis was conducted by the COVID-19 Genomic Surveillance Network in Japan (COG-JP), overseen by the National Institute of Infectious Diseases. The SARS-CoV-2 present in the BALF was suspected to be the Omicron variant (XBB.1.16).

**Figure 4:**
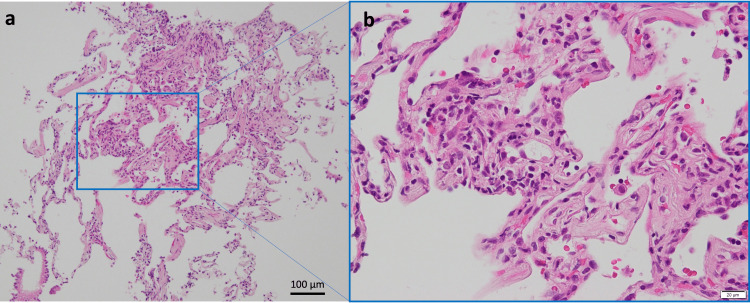
Histological examination of TBLB. (a) Hematoxylin and eosin staining of TBLB from the right B^2^b, B^3^a, and B^8^b revealing the presence of infiltrative lymphocytes and neutrophils in the alveolar interstitium. Neither fibrosis nor granulation tissue buds were observed. (b) Higher magnification. TBLB, transbronchial lung biopsy

Based on the clinical course, radiological findings, and the detection of SARS-CoV-2 in BALF, the diagnosis of persistent COVID-19 pneumonia was confirmed. Treatment with 10 g of intravenous immunoglobulin supplementation and RDV (an initial dose of 200 mg followed by daily doses of 100 mg) was initiated post-bronchoscopy. By day 5, the patient exhibited marked improvement in general fatigue, and late inspiratory crackles had resolved. On day 8, TBLB pathology results were received, revealing the presence of infiltrative lymphocytes and neutrophils in the alveolar interstitium (Figure 4).

Neither fibrosis nor granulation tissue buds were observed, further corroborating the diagnosis of COVID-19 pneumonia. On day 11, with chest CT images showing improvement of patchy ground-glass opacities in both lungs and a CRP level decrease to 0.16 mg/dL, RDV was discontinued. Following persistent hypogammaglobulinemia with a serum IgG level of 660 mg/dL, an additional 10 g of intravenous immunoglobulin supplementation was administered. The patient continued to recover and was discharged on day 14.

## Discussion

We experienced a case of persistent COVID-19 pneumonia resembling COP in a patient with lymphoma remission. The clinical question centered on whether the patient's current COVID-19 pneumonia was a recurrence or a re-infection by a different SARS-CoV-2 variant. This was prompted by (i) literature indicating COVID-19 recurrence in immunocompromised patients and (ii) the persistence of ground-glass opacities on chest CT from her prior COVID-19 pneumonia. Therefore, we sent BALF samples to the public health center to identify the variant. As a result, the Omicron variant (XBB.1.16) was suspected. Given the major variant influenced by this admission, we concluded that COVID-19 pneumonia was a re-infection rather than a recurrence. If earlier prevalent variants like Delta (B.1.617.2) had been detected, casirivimab-imdevimab could have been a treatment consideration [14].

Similar to the present patient, a case report describes a lymphoma patient treated with rituximab who presented with COVID-19 pneumonia with chest CT scans revealing bilateral ground-glass opacities. The nasal swab PCR was negative, but the BALF PCR was positive [15]. Such cases highlight the diagnostic utility of BAL for COVID-19 pneumonia, especially when nasopharyngeal swabs yield negative results. Another study found that 32 of 198 patients initially testing negative via nasopharyngeal swabs were subsequently positive via BAL [16]. Considering that BALF specimens have the highest positivity rate (93%), compared to sputum (72%) and nasal swabs (63%), BALF remains a viable diagnostic option for suspected COVID-19 pneumonia, even when nasal swab results are negative [17].

In this patient, both the BioFire FilmArray Respiratory Panel 2.1 and the Smart Gene® SARS-CoV-2 Detection Kit detected SARS-CoV-2 in the BALF. The manufacturer recommends these methods for nasopharyngeal swabs, leaving their efficacy largely unverified for BALF. A retrospective study employing the FilmArray Respiratory Panel 2.1 method on immunocompromised patients reported the detection of pathogens like SARS-CoV-2 and adenovirus in isolated cases out of 23. To address (i) the potential dilution of SARS-CoV-2 viral particles in BALF and (ii) evidence of viral infection in alveolar cavity immune cells, BALF was concentrated approximately 50-fold before testing [18]. This process equates to analyzing about 1.5x10^6^ cells. Considering the negative SARS-CoV-2 antigen and low viral RNA levels in the BALF, it is inferred that few infectious viral particles exist, yet SARS-CoV-2 might continuously infect lung cells, leading to sustained inflammation.

Regarding BALF lymphocytosis, 15% of ventilated COVID-19 patients have shown lymphocyte levels over 20% [18]. Notably, increased T-cells and monocytes characterize BALF from mechanically ventilated COVID-19 patients [19]. A patient with lymphoma undergoing rituximab therapy manifested a 35% lymphocytosis in BALF, akin to the current case [15]. Although comprehensive data on mild COVID-19 pneumonia are scarce, these instances suggest BALF lymphocytosis is possible. If the SARS-CoV-2 gene test had not been conducted in BALF, the patient might have been misdiagnosed with COP based on imaging and BALF lymphocytosis. We found a decreased CD4/CD8 ratio of 0.11, with an increased HLA-DR+CD3+ population up to 72.8% in BALF. This increased HLA-DR+CD3+ population was concordant with the previous study that found that increased levels of HLA-DR+CD16+CD3+ T cells were detected in BALF from COVID-19, and these cells play a pathological role in severe COVID-19 [20].

We also experienced another patient who exhibited similarities to the present case with an instructive clinical course. After completing obinutuzumab maintenance therapy for lymphoma, this patient contracted and recovered from COVID-19. Six months later, the patient developed pneumonia with organizing pneumonia-like shadows. With negative results for nasopharyngeal SARS-CoV-2 antigen and PCR, the patient was clinically diagnosed with organizing pneumonia and treated with corticosteroids. This treatment initially helped, but the patient's condition eventually deteriorated, with subsequent tests confirming SARS-CoV-2. This highlights the need for considering persistent SARS-CoV-2 infection, especially in immunocompromised patients, even when nasopharyngeal tests are negative.

## Conclusions

In immunocompromised patients, COVID-19 pneumonia can manifest similarly to organizing pneumonia. The presented case underscores the diagnostic significance of BAL when nasopharyngeal swabs for SARS-CoV-2 are negative. Relying exclusively on nasopharyngeal swabs can lead to misdiagnosis and potentially detrimental treatments.
